# High-Temperature Oxidation Behavior of TiB_2_-HfB_2_-Ni Cermet Material

**DOI:** 10.3390/ma15248860

**Published:** 2022-12-12

**Authors:** Zhuo Wang, Jiaojiao Gao, Jinpeng Song

**Affiliations:** 1 College of Mechanical and Vehicle Engineering, Taiyuan University of Technology, Taiyuan 030024, China; 2 College of Aeronautics and Astronautics, Taiyuan University of Technology, Taiyuan 030024, China

**Keywords:** TiB_2_-HfB_2_-Ni cermet, oxidation behavior, high temperature, microstructure

## Abstract

To analyze the high-temperature oxidation behavior of TiB_2_-HfB_2_-Ni cermet material, TiB_2_-HfB_2_-Ni cermets were fabricated by hot-pressing sintering technology. The oxidation resistance and the thermal fracture of TiB_2_-HfB_2_-Ni cermet were investigated at 1100 °C for 1, 4, 7, and 10 h, respectively. Before oxidation, TiB_2_-HfB_2_-Ni cermet, consisting of TiB_2_, HfB_2_, and Ni, had the core-rim structure. The core was TiB_2_ grain and the rim was composed of Ni and solid solution (Ti, Hf)B_2_. After oxidation at 1100 °C, the oxides of the TiB_2_-HfB_2_-Ni cermet were mainly TiO_2_, HfO_2_, B_2_O_3_, and NiO, which the oxidation process abided by the parabolic law. With the oxidation time increasing from 1 h to 10 h, the oxidation degree of the TiB_2_-HfB_2_-Ni cermet increased, and the oxide layer became thicker. The oxide layer was thin and dense after oxidation at 1100 °C for 1 h. An obvious boundary was discovered between the transition layer and the substrate layer after oxidation at 1100 °C for 7 h. The thermal fracture occurred in the contact regions of different layers at 1100 °C for 10 h. TiB_2_-HfB_2_-Ni took place in oxidation at different levels from the outer to the inner, and the components of different oxide layers were certainly distinct.

## 1. Introduction

TiB_2_-based cermet demonstrates excellent mechanical, physical, and chemical properties, including high hardness at high temperatures, high melting point, outstanding electrical conductivity, and strong corrosion resistance [1,2,3,4]. TiB_2_-based cermet materials (TiB_2_ + BN [4], TiB_2_ + W [5], TiB_2_ + HfC [6], TiB_2_ + Si_3_N_4_ [7] and TiB_2_ + TiC [8]) were used in armored parts, high-temperature components, and cutting tools. Since TiB_2_-based cermets were usually served in high-temperature oxidation environments [9,10,11,12], it was of great significance to investigate the high-temperature oxidation behavior for long-term use.

Recently, some researchers have investigated the oxidation behavior of TiB_2_-based cermets at different temperatures. The oxides TiO_2_ and B_2_O_3_ were discovered in the monolithic TiB_2_ cermet oxidized at 800−1200 °C, and the oxide layer thickness, oxide content, and microstructure were affected by the oxidation temperature and time [13,14,15]. TiB_2_-WSi_2_ cermet demonstrated better oxidation resistance than TiB_2_ cermet in the same high-temperature environment, and oxides TiO_2_, B_2_O_3_, and SiO_2_ formed on the surface of TiB_2_-WSi_2_ cermet [16]. The oxide layer thickness of the TiB_2_ + EuB_6_ cermet was about 340 μm during oxidization at 1400 °C for 8 h, and the oxides could enhance the oxidation resistance of the cermet [17]. The glassy B_2_O_3_ was discovered in TiB_2_-MoSi_2_-CrB_2_ cermet and TiB_2_-SiC-B_4_C cermet oxidized at 1000 °C [18,19].

Moreover, the relationship between oxidation duration and mass gain of TiB_2_-based cermet was investigated by some researchers. The relationship between the oxidation duration and the mass gain of TiB_2_ + TiC cermet abided by the linear law at 600 °C, while abided by the parabolic law at 700 °C and 800 °C [20]. TiB_2_ cermet coating could improve the oxidation temperature by up to 900 °C for molybdenum, and the oxidation behavior abided by the parabolic law [21]. The oxidation kinetics of TiB_2_ and TiB_2_ + MoSi_2_ also abided by the parabolic law at 850 °C [22].

To investigate the oxidation mechanism of the TiB_2_-HfB_2_-Ni cermet in high temperatures, TiB_2_-HfB_2_-Ni cermets would be fabricated by hot-pressing sintering technology. The microstructures before and after oxidation and the high-temperature oxidation behavior of TiB_2_-HfB_2_-Ni cermets would be investigated.

## 2. Experimental Procedures

Commercially available TiB_2_, HfB_2_, and Ni powders (>99.9%, 1 μm) were utilized as raw powders to fabricate TiB_2_-HfB_2_-Ni cermet (TBHB). The mixed powders (72 wt.% TiB_2_, 20 wt.% HfB_2_, and 8 wt.% Ni), after milling with WC balls and ethanol absolute for 72 h, drying and sieving orderly, were put into the graphite mold and then sintered at 1500 °C for 0.5 h under an axial pressure of 35 MPa in the vacuum sintering furnace (ZT-40-20, Shanghai Chenhua Technology Co., Ltd., Shanghai, China). The sintered ceramics were machined into strip specimens with a dimension of 3 mm × 4 mm × 40 mm and surface roughness of 0.96 μm. More details about the fabricating process can be found in the literature [6,23].

To investigate the oxidation behavior of TBHB, first, the TBHB specimens were cleaned ultrasonically and dried. The lengths, widths, and heights of the TBHB specimens before oxidation were measured using Vernier caliper (100011956107, Shanghai Tool Works Co., Ltd., Shanghai, China, accuracy 0.02 mm) and the surface areas (*S*) were calculated. The weights (*m*_0_) were measured using an electronic balance (BSM-120.4, Shanghai Zhuojing Electronic Technology Co., Ltd., Shanghai, China, accuracy 0.1 mg). Second, the TBHB specimens leaned on the wall of the corundum crucible-arc to reduce the contact area. Then, the corundum crucible-arc was put into the muffle furnace (ZDXS5-2.5-120, Shenzhen Zhongda Strong Electric Furnace Co., Ltd., Shenzhen, China). The TBHB specimens were oxidized at 1100 °C for 1, 4, 7, and 10 h, respectively. The oxidation experiments were completed under static lab air. Finally, the weights (*m*_1_) of the TBHB specimens after oxidation were measured. The mass gain per unit area (Δ*m*/*S*) was calculated as follows:

Δ*m*/*S* = (*m*_1_ − *m*_0_)/*S* (1)

More than 5 specimens were tested to calculate the average. X-ray diffraction (XRD EMPYREAN, PANalytical B.V., Almelo, The Netherlands) and Energy dispersive spectrometer (EDS, ACT-350, Oxford Instruments, Oxford, UK) were employed to measure the compositions of the sintered TBHB and the oxidized TBHB samples. The scanning electron microscope (SEM, Supra-55, Carl Zeiss AG, Jena, Germany) was used to analyze the polished surfaces and fracture morphologies of the TBHB specimens.

## 3. Results and Discussions

### 3.1. Microstructure of TBHB before Oxidation

**Figure 1:**
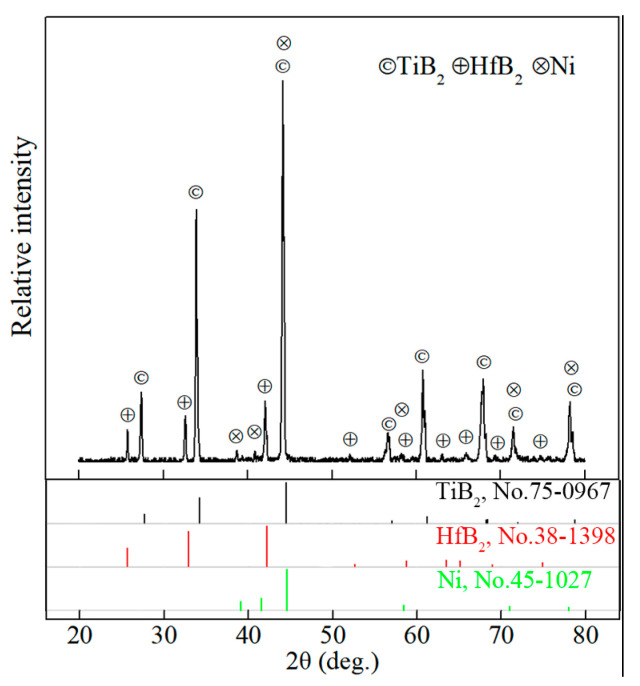
XRD pattern of TBHB before oxidation.

XRD pattern of TBHB before oxidation is shown in Figure 1. TBHB consisted of TiB_2_, HfB_2_, and Ni. It demonstrated that no reaction occurred in the sintering process and Ni had good chemical compatibility with TiB_2_ and HfB_2_. The literature [5] reported that a small amount of Ni_4_B_3_ was discovered in the TiB_2_-Ni system, and brittle material Ni_4_B_3_ was harmful to the mechanical properties of TiB_2_-based ceramics. However, the Ni_4_B_3_ was not discovered in TBHB according to Figure 1.

**Figure 2:**
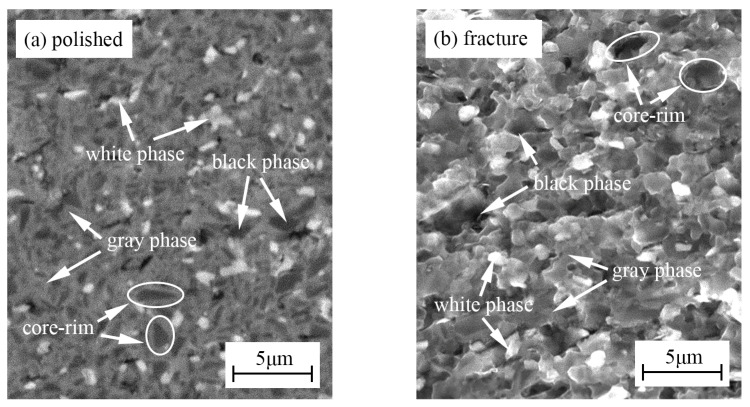
Polished surface and fracture morphology of TBHB before oxidation: (**a**) polished surface, (**b**) fracture morphology.

**Figure 3:**
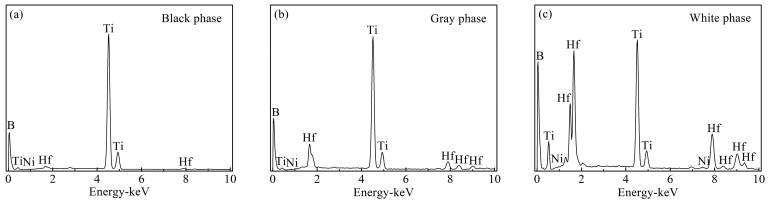
EDS results of the phases in TBHB before oxidation: (**a**) black phase, (**b**) gray phase, (**c**) white phase.

Figure 2 shows the polished surface and fracture morphology of TBHB before oxidation. There were mainly three phases including a black phase, a gray phase, and a white phase. Figure 3 shows the EDS results of three phases in TBHB before oxidation. Based on the results of XRD and EDS, the black phase was the undissolved TiB_2_ with abundant Ti, and scant Hf, B, and Ni, as shown in Figure 3a. The gray phase was the solid solution (Ti, Hf)B_2_ rich in Ti, but poor in Hf, B, and Ni, as shown in Figure 3b. The white phase was the solid solution (Ti, Hf)B_2_ rich in Ti, Hf, and B, but poor in Ni. In addition, the core-rim structure was discovered in the polished surface and fracture morphology [6]. The core was TiB_2_ grain and the rim was composed of Ni and the solid solution (Ti, Hf)B_2_. The elements Ti and Hf, belonging to the same family, have the same valence as B for TiB_2_ and HfB_2_. In the liquid sintering process; therefore, some Ti and Hf atoms were probably exchanged to form (Ti, Hf)B_2_ solid solution and Ni was often distributed among TiB_2_ grains [24]. The solid solution and Ni around the TiB_2_ grains formed a skeleton structure in TBHB, which could keep the matrix from fracturing when TBHB undertook high load or was oxidized at a high-temperature environment. In addition, the shapes of TiB_2_ grains would change from blocky to banded structures, which might result from the mutual replacement of Ti and Hf. The banded structure could enhance the mechanical properties of TiB_2_-based cermets [6,23].

### 3.2. Oxides and Mass Gain in TBHB

**Figure 4:**
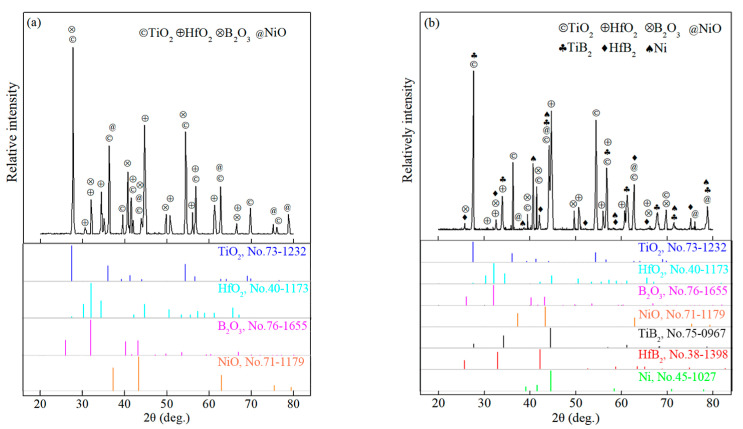
XRD patterns of TBHB surface and cross-section after oxidation at 1100 °C for 10 h: (**a**) surface, (**b**) cross-section.

Figure 4 shows the XRD patterns of the TBHB surface and cross-section after oxidation at 1100 °C for 10 h. There were oxides TiO_2_, HfO_2_, B_2_O_3_, and NiO on the surface of TBHB, as shown in Figure 4a. However, aside from the above oxides, there was TiB_2_, HfB_2_, and Ni on the cross-section of TBHB, as shown in Figure 4b. This indicated that the inside of TBHB were incompletely oxidized.

The following possible reactions occurred during the oxidation process.

Ni(s) + 0.5O_2_(g) → NiO(s) (2)

TiB_2_(s) + 2O_2_(g) → TiO(s) + B_2_O_3_(l) (3)

TiB_2_(s) + 2.5O_2_(g) → TiO_2_(s) + B_2_O_3_ (4)

HfB_2_(s) + 2.5O_2_(g) → HfO_2_(s) + B_2_O_3_ (5)

2TiB_2_(s) + 4.5O_2_(g) → Ti_2_O_3_(s) + 2B_2_O_3_(l) (6)

B_2_O_3_(l) → B_2_O_3_(g) (7)

According to reactions (3), (4), and (6), the possible oxides of TiB_2_ were TiO, Ti_2_O_3_, TiO_2_, and B_2_O_3_. However, the obvious peaks of TiO and Ti_2_O_3_ were undiscovered in the XRD of the oxidation TBHB. This is because low-valence oxides TiO and Ti_2_O_3_ were easily oxidized to TiO_2_ [25]. Therefore, TiO_2_ and B_2_O_3_ were the main oxides of TiB_2_ in the TBHB. In the oxidizing process, the oxides NiO, TiO_2_, and HfO_2_ were solid because their melting points were far higher than 1100 °C. B_2_O_3_ was liquid with a high viscosity because its melting point (450 °C) was lower than 1100 °C. The B_2_O_3_ liquid filled the pores among the oxide particles at 1100 °C, which could prevent oxygen from eroding the interior materials and transforming into glassy after cooling [19]. Moreover, the B_2_O_3_ liquid evaporated, which would weaken the protective effect of B_2_O_3_ on the cermet [16].

**Figure 5:**
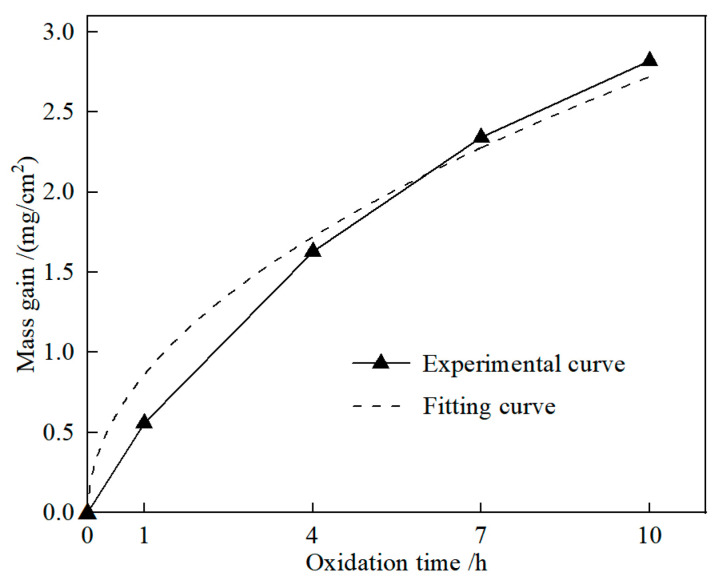
Experimental and fitting curve of oxidation duration and mass gain of TBHB at 1100 °C.

Reactions (2)−(6) would result in the mass of TBHB increasing, while reaction (7) would result in the mass decreasing. Figure 5 shows the experimental and fitting curve of oxidation duration and mass gain of TBHB at 1100 °C. As the oxidation time extended, the mass gain increased gradually. When the oxidation times were 1, 4, 7, and 10 h, the values of mass gain of TBHB were 0.56, 1.63, 2.34, and 2.82 mg/cm^2^, respectively. The fitting curve was derived from the experimental data and the equation was expressed as [20]:

(∆*m/S*)^2^ = 0.74*t* (8)

where *m* and *t* were the mass gain and the oxidation time, respectively. Equation (8) is a parabolic equation. According to Figure 5, the fitting curve agreed with the experimental curve well, which indicated that the oxidation process of TBHB abided by the parabolic law.

**Figure 6:**
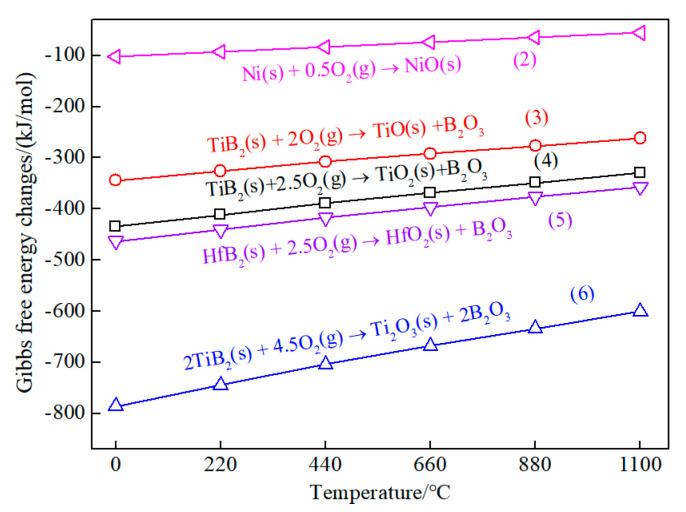
Variations of the Gibbs free energy for the oxidation reactions of Ni, TiB_2_, and HfB_2_ at 0−1100 °C.

Figure 6 shows the Gibbs free energy changes of the oxidation reactions of Ni, TiB_2_, and HfB_2_ at 0–1100 °C. The Gibbs free energies were calculated using the HSC-chemistry software. Generally, the change of the Gibbs free energy was used to estimate the tendency of the reactions at the same oxidation condition [26,27,28]. The lower the value of the Gibbs free energy, the stronger the reaction tendency. According to Figure 6, the ranking of the reaction tendency was (2) < (3) < (4) < (5) < (6) at 0–1100 °C. Since the outer layer was in direct contact with oxygen, the compounds on the TBHB surface might are oxidized into the oxides such as TiO, Ti_2_O_3_, TiO_2_, HfO_2_, B_2_O_3_, and NiO. However, low-valence titanium ions (Ti^2+^ and Ti^3+^) would be further oxidized to high-valence Ti^4+^ in the atmosphere [25]. Therefore, the final oxides on the surface TBHB surface were TiO_2_, B_2_O_3_, and NiO.

The oxidation of the materials in the TBHB inner was controlled by the oxygen diffusion speed. When the oxygen entered into the TBHB inner, TiB_2_, HfB_2_, and Ni would be competitive with each other for oxygen to form oxides. According to the reaction tendency, TiB_2_ firstly reacted with oxygen to form oxides Ti_2_O_3_ and B_2_O_3_, and low-valence oxide Ti_2_O_3_ would be oxidized to TiO_2_. Then, HfO_2_ and Ni would be oxidized orderly if there was enough oxygen to maintain the reactions. When the B_2_O_3_ liquid formed and filled the pores, the oxygen diffusion would gradually slow down, resulting in oxides decreasing in the TBHB inner. Thus, the oxide content reduced gradually from the outer to the inner. The oxides and the unreacted materials would constitute new composites or mixtures with their physical properties.

### 3.3. Thermal Fracture of TBHB in the High-Temperature Oxidation Process

**Figure 7:**
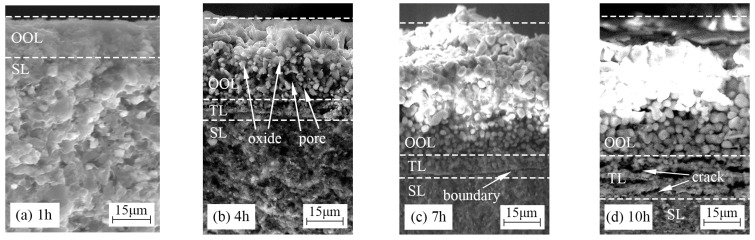
Fracture morphologies of TBHB at 1100 °C in different oxidation times: (**a**) 1 h, (**b**) 4 h, (**c**) 7 h, (**d**) 10 h.

Figure 7 presents the fracture morphologies of TBHB oxidized at 1100 °C for 1, 4, 7, and 10 h. The oxidation of TBHB became more severe and the oxide layer became thicker as the oxidation time extended from 1 h to 10 h. In Figure 7a, the oxide layer, consisting of the outer oxide layer (OOL), was about 15 μm at 1100 °C for 1 h. The thin and dense OOL could inhibit the entrance of oxygen into the TBHB inner part. In Figure 7b, the oxide layer, consisting of OOL and the transition layer (TL), was about 30 μm at 1100 °C for 4 h. OOL was composed of oxide particles and pores. The growth of oxide and the evaporation of B_2_O_3_ liquid lead to pores. The connected pores in the OOL formed convenient channels for oxygen to enter into the TBHB inner and erode the inner materials, and then the dense TL formed in the TBHB inner. In Figure 7c, the oxide layer, consisting of OOL and TL, was about 50 μm at 1100 °C for 7 h. An apparent boundary between the dense TL and substrate layer (SL) could be observed. In Figure 7d, the oxide layer, consisting of OOL and TL, was about 70 μm at 1100 °C for 10 h. Cracks were observed in TL, which indicated that the thermal fracture took place in the contact layers between the OOL and TL or TL and SL. Moreover, the size of the oxide particles from the outer to the inner gradually decreased as shown in Figure 7. This indicated that TBHB took place in oxidation at different levels from the outer to the inner.

**Figure 8:**
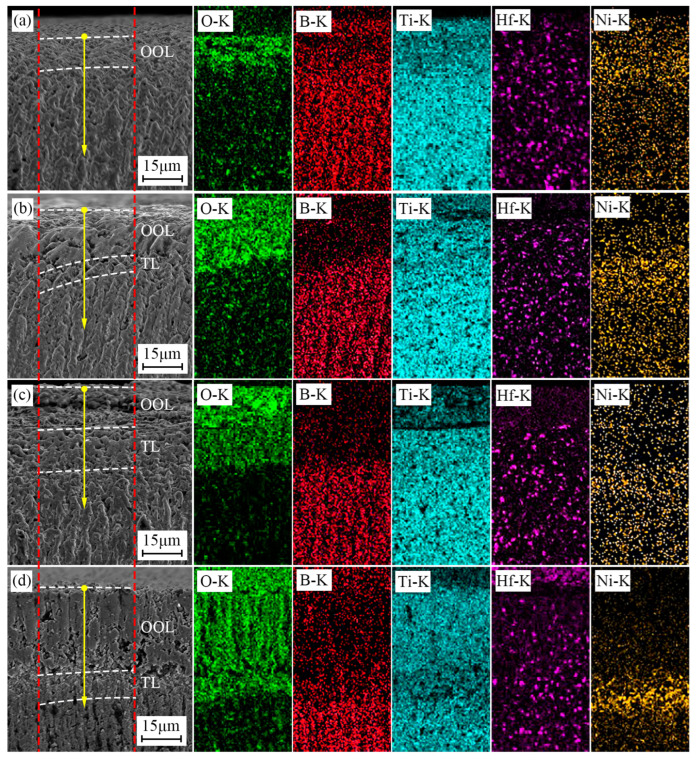
BSE patterns and the corresponding EDS map scan results of cross-section of TBHB at 1100 °C in different oxidation times: (**a**) 1 h, (**b**) 4 h, (**c**) 7 h, (**d**) 10 h.

Figure 8 shows the BSE patterns and the corresponding EDS map scan results of TBHB cross-sections at 1100 °C in different oxidation times. The scanned area was the gap between two red dotted lines. In Figure 8, the concentrations of O elements in OOL and TL were more than in SL, and B element was less than in SL. This was because the oxidation reactions of TiB_2_, HfB_2_, and Ni occurred at 1100 °C, which resulted in an increase in O element. The B_2_O_3_ liquid in the reactions (3)–(6) evaporated, which resulted in a decrease in the B element. There were no significant concentration changes in Ti, Hf, and Ni elements.

**Figure 9:**
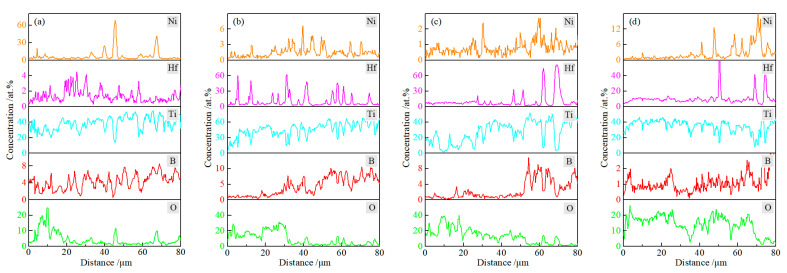
Line EDS results of cross-section of TBHB at 1100 °C in different oxidation times: (**a**) 1 h, (**b**) 4 h, (**c**) 7 h, (**d**) 10 h.

Figure 9 presents the line EDS results of TBHB cross-sections at 1100 °C for 1, 4, 7, and 10 h. The paths of line EDS were shown in the yellow arrows in Figure 8. From the edge to the center of TBHB, the concentrations of O and B elements changed significantly and regularly. When the oxidation times were 1, 4, 7, and 10 h, the concentration of O element increased or B element decreased at a distance of about 15, 30, 50, and 70 μm, respectively. When the oxidation time was 1 h, the content of B element did not change significantly, which was due to the insufficient evaporation of B_2_O_3_ in a short time. B_2_O_3_ liquid densified the oxide layer after cooling (as shown in Figure 7a). When the oxidation times were 4, 7, and 10 h, the evaporation of B_2_O_3_ resulted in obvious pores in OOL. The extreme difference in oxide content resulted in the thermal fracture of OOL and TL or TL and SL, and the cracks were observed in TL (as shown in Figure 7c,d). According to the variations of O and B elements, therefore, TBHB took place in oxidation at different levels from the outer to the inner, and the components of OOL, TL, and SL were certain differences.

## 4. Conclusions

TiB_2_-HfB_2_-Ni cermets were papered by hot-pressing sintering. The microstructures and oxidation mechanism of the TiB_2_-HfB_2_-Ni cermets were investigated. The conclusions were as follows:

(1) Before oxidation, TiB_2_-HfB_2_-Ni cermets, consisting of TiB_2_, HfB_2_, and Ni phases, had the core-rim structure. The core was TiB_2_ grain and the rim was composed of Ni and the solid solution (Ti, Hf)B_2_.
(2) After oxidation, the oxides of TiB_2_-HfB_2_-Ni cermet surface were TiO_2_, HfO_2_, B_2_O_3_, and NiO. Aside from the above oxides, there was TiB_2_, HfB_2_, and Ni on the cross-section. The values of mass gain at 1100 °C for 1, 4, 7, and 10 h were 0.56, 1.63, 2.34, and 2.82 mg/cm^2^, respectively. The oxide content reduced gradually from the outer to the inner of TiB_2_-HfB_2_-Ni cermet. With the oxidation time increasing from 1 h to 10 h, the oxidation of TiB_2_-HfB_2_-Ni cermet surface became more severe and the oxide layer became thicker.
(3) The thicknesses of the oxide layers at 1100 °C for 1, 4, 7, and 10 h were about 15, 30, 50, and 70 μm, respectively. The oxide layer was thinner and dense at 1100 °C for 1 h. An obvious boundary was observed between the transition layer and the substrate layer at 1100 °C for 7 h. The thermal fracture occurred in contact regions of different layers at 1100 °C for 10 h. TBHB took place in oxidation at different levels from the outer to the inner, and there were some differences between the components of the outer oxide layer, transition layer, and substrate layer.

## Data Availability

Not applicable.
